# Data science and artificial intelligence for maternal, newborn and child health: scoping review and thematic analysis

**DOI:** 10.1186/s12889-025-25430-0

**Published:** 2025-11-28

**Authors:** Joseph Akuze, Grieven P. Otieno, Samson Yohannes Amare, Bancy Ngatia, Phillip Wanduru, Fati Kirakoya-Samadoulougou, Rornald Muhumuza Kananura, Agbessi Amouzou, Abiy Seifu Estifanos, Eric O. Ohuma

**Affiliations:** 1 Faculty of Epidemiology and Population Health, London School of Hygiene & Tropical Medicine, London, UK; 2 Centre of Excellence for Maternal Newborn and Child Health Research, Makerere University School of Public Health, Kampala, Uganda; 3 Kenya Paediatric Research Consortium, Nairobi, Kenya; 4 Mekelle University, Mekelle, Ethiopia; 5 Department of Global Public Health, Karolinska Institute, Stockholm, Sweden; 6 Centre de Recherche en Epidémiologie, Biostatistique et Recherche Clinique, Université Libre de Bruxelles, Brussels, Belgium; 7 Department of International Health, Johns Hopkins Bloomberg School of Public Health, Baltimore, MD USA; 8 African Population and Health Research Center, Nairobi, Kenya; 9 Centre for Implementation Sciences (CIS), School of Public Health, Addis Ababa University, Addis Ababa, Ethiopia; 10 Department of Reproductive, Family, and Population Health, School of Public Health, Addis Ababa University, Addis Ababa, Ethiopia

**Keywords:** Data science, Artificial intelligence, Maternal health, Newborn health, Child health, Africa, Scoping review

## Abstract

**Introduction:**

In 2020, an estimated 287,000 women died in pregnancy or childbirth-related causes (70% in Africa), while 2.3 million newborns died in the first 28 days of life in 2022 (46% in Africa). The utility of data science and Artificial Intelligence (AI) in health has potential to contribute and accelerate innovation, improve data use for evidence-based decision making, and better planning for policy decision making.

**Aim:**

To map the current landscape of data science and artificial intelligence applications in maternal, newborn and child health (MNCH) across Africa and identify gaps, challenges, and opportunities for future implementation.

**Methods:**

A scoping review was conducted following the Arksey and O’Malley framework across five databases (PubMed, Web of Science, EMBASE, SCOPUS, Ovid) and grey literature published before December 2023. Thematic analysis was conducted using previously published seven-domain framework to identify patterns in data science applications, challenges and opportunities. Additionally, projects within the maternal newborn and child health focus on the WHO’s Digital Health Initiatives Atlas were reviewed.

**Results:**

Of 11,320 articles screened, 52 articles from 31 African countries met inclusion criteria. Most studies (*n* = 34, 65.4%) were from Eastern Africa. Most studies were research projects (*n* = 28, 53.8%) and demographic health surveys (*n* = 22, 43.1%) rather than operational implementation government programmes. Key themes identified included infrastructure challenges, data quality issues, limited workforce capacity, and heavy reliance on external funding. The WHO’s Digital Health Atlas revealed 659 data science initiatives across Africa with the earliest recorded in 2002. 316 (48%) projects were focussed on MNCH and were implemented in 44 African countries.

**Conclusion:**

The limited application of data science and artificial intelligence in MNCH at the national level highlights a significant gap in Africa. Our review found that 28 studies (53.8%) were research projects compared to operational implementations integrated into routine systems to inform policy. Main barriers include inadequate infrastructure, limited data stewardship capacity, and insufficient government commitment. Opportunities exist through Africa’s youthful demographics, expanding mobile technology, and latecomer advantage in digital innovation.

**Supplementary Information:**

The online version contains supplementary material available at 10.1186/s12889-025-25430-0.

## Introduction

Data science is defined as “an interdisciplinary field of inquiry that merges scientific methodologies, algorithms, and systems to analyse both structured and unstructured data” [1]. Data science uses quantitative and analytical approaches, processes and systems to extract knowledge and insights from large and complex datasets and encompasses several topics, notably: big data, analytics, artificial intelligence, machine learning, digital technologies, informatics, modelling, mobile technologies and visualisation [2, 3].

In the maternal, newborn, and child health (MNCH) context, data science has the potential to transform our understanding and utilisation of health data for example in diagnosing and classifying newborn conditions, gestational age estimation, predicting adverse pregnancy outcomes and pattern recognition, ultimately leading to improved health outcomes [4]. As an extension of data science, artificial intelligence has increasingly gained recognition globally for its applicability to various disciplines including maternal, newborn and child health (MNCH) [5, 6]. Reliable health information systems are essential for tracking MNCH indicators related to global initiatives such as Sustainable Development Goals (SDGs) [7, 8], Every Woman Every Newborn Everywhere (EWENE) initiative (previously known as Every Newborn Action Plan (ENAP)) [9], and the Ending Preventable Maternal Mortality (EPMM) initiative [10].

Despite several sub-Saharan African (SSA) countries demonstrating improvements in vaccine coverage, nutrition, healthcare access, family planning and skilled birth attendance since the Millennium Development Goals (MDGs) between 1990-to-2015 [11, 12], SSA bears the highest burden for stillbirths (47% of the global 1.9 million stillbirths) and neonatal deaths (46% of the 2.3 million preventable neonatal deaths) [13, 14]. Since the MDGs era, slow progress has been observed in the rate reduction for stillbirth (2.3% per year), neonatal death rates (2.9% per year), under-five child deaths (4.3% per year) and maternal mortality (4.2% per year) [13, 15, 16], due to poor quality of care during pregnancy and during/at birth; limited preventive interventions and health workforce; inadequate social recognition of stillbirths’ impact on families [17]; measurement and data gaps challenges [18].

National data from African countries are often missing, incomplete, or of poor quality, thereby limiting the ability to monitor key health indicators [19, 20]. SSA countries rely on periodic household surveys like Demographic and Health Survey (DHS) and Multiple Indicator Cluster Surveys (MICS) to assess progress made towards international goals such as SDGs [21, 22]. The key barriers to robust data collection and utilisation include inadequate infrastructure, funding, inefficient management and operation of the health systems, inadequate and inequitable distribution of health workforce, poor planning and budgeting [23, 24]. In addition, most SSA countries do not have nationally implemented electronic health records. These shortcomings lead to non-evidence-based decision-making, hinder access to quality MNCH care and adverse outcomes [25].

To improve MNCH outcomes in Africa, it is essential to incorporate data science and Artificial Intelligence in health to help accelerate innovation and improve data use for evidence-based decision making, identification of where gaps lie for prioritisation of interventions, and better planning for policy [5, 12, 26]. Achieving this goal will require multifaceted collaborative efforts aimed at building and strengthening capacity, improving data infrastructure and data quality, increasing demand for data use across all levels of the health system, and developing robust information systems along with sound data governance and policies.

There is a significant opportunity to capitalise on Africa’s advancing digital infrastructure and internet connectivity, which have enabled the overwhelming success of financial technology entities such as mobile money services [27]. The rapid growth of mobile health technology (m-health) in Africa has resulted in the collection of vast amounts of data that will require advanced data science and artificial intelligence approaches to process and analyse. Evidence suggests that mobile health interventions can significantly enhance health service utilisation among women in the perinatal period [6, 28]. Therefore, highlighting the potential role that technology, data science, and artificial intelligence can play in accelerating progress towards MNCH targets and improving health outcomes is key.

Despite the increasing relevance and application of data science to health data in Africa, the specific impact on MNCH remains largely unexplored in SSA [19, 29]. This paper seeks to illuminate the extent to which data science and artificial intelligence has been utilised to solve key MNCH issues in Africa, explore any potential challenges and gaps hindering the wide application of data science for MNCH priorities, and explore the untapped potential that could be a contributor in accelerating data science applications for health in Africa. To achieve this, we follow a three-pronged approach. First, a scoping review to comprehensively map the existing landscape of data science applications in MNCH across Africa. Second, we analyse data on projects registered on the WHO Digital Health Initiatives Atlas and any relevant data science examples from Africa as case studies focusing on those utilising data science for MNCH in Africa [4]. We evaluate this systematically based on our previously published framework [4] encompassing seven domains: (i) Infrastructure and Systemic Challenges, (ii) Data Quality, (iii) Data Governance, Regulatory Dynamics and Policy, (iv) Technological Innovations and Digital Health, (v) Capacity Development, Human Capital and Opportunity (vi) Collaborative and Strategic Frameworks, and (vii) Recommendations for Implementation and Scaling (Supplementary Fig. 1). Finally, we synthesise the findings from the scoping review, WHO Digital Health Initiatives Atlas review, and case studies from Africa to highlight key issues and emerging themes, challenges and opportunities, offering insights into the future trajectory of data science in MNCH and provide recommendations for its effective implementation and scale-up.

## Methods

Our scoping review protocol was registered on the Open Science Framework (OSF) [4] and is published in BMJ Open [4]. To investigate and synthesise the transformative potential of data science in MNCH in Africa, we employed a two-pronged methodological approach: a summary and thematic analysis of scoping review process (Section 1a and 1b respectively), an overview and thematic analysis of data from the Digital Health Atlas (Section 2a and 2b respectively) and a discussion of results from the case studies and the overall landscape of data science in Africa. Each section was reviewed separately by a different reviewer. This approach allowed us to identify the existing gaps, challenges, opportunities and to offer recommendations for enhancing and integrating data science in MNCH initiatives.

### Section 1a: scoping review summary

We conducted a scoping review based on the methodological framework developed by Arksey and O’Malley [30] to explore the utilisation of data science in addressing MNCH priority questions in Africa. This framework was chosen due to the evolving nature of data science in Africa and its limited documentation, details of which are presented in our scoping review protocol [4].

#### Operationalisation of Arskey and O’malley framework for scoping reviews

Following the five stages of the Arksey and O’Malley framework [30], we (1) identified research questions focussed on data science applications in MNCH across Africa; (2) identified relevant studies through systematic database and website searches; (3) selected studies based on predetermined inclusion and exclusion criteria and (4) collated and summarised results through thematic analyses using our seven-domain framework that was published in our protocol paper [4].

As outlined in our published protocol, our scoping review focus is maternal, newborn, child health populations in African countries (Population), data science and artificial intelligence applications notably, machine learning, big data analytics, and digital health technologies (Concept) and African healthcare settings including clinical, community and national health system levels across all 54 African countries (Context), therefore using a population concept and context framework.

#### Data sources and search strategy

A comprehensive literature search was performed across PubMed, Web of Science, EMBASE, SCOPUS and Ovid. We also searched the reference lists, reports, and grey literature sources including WHO and African Union reports, government publications and organisational websites (Data Science for Health Discovery and Innovation in Africa (DS-I Africa), WHO’s Digital health atlas and Data Science Africa). The search terms focussed on the intersection of data science and MNCH components (maternal health, neonatal health, child health and perinatal) within the African context (All 54 African countries were eligible for inclusion). Key search terms included combinations of: ‘data science,’ ‘artificial intelligence,’ ‘machine learning,’ ‘maternal health,’ ‘newborn,’ ‘child health,’ and African country names. The search covered publications from inception to December 2023. Boolean operators “OR” and “AND” were used. Our complete search strategy is provided in Supplementary Table S1 for detailed search terms.

#### Study screening and selection

The screening and selection process involved several steps: (a) removal of duplicates, (b) review of the titles, (c) review of the executive summaries or abstract, and (d) review of full texts. Three reviewers were involved in the study screening process. Disagreements between reviewers were resolved through discussion, with a third reviewer (EOO) consulted when consensus could not be reached.

#### Inclusion and exclusion criteria

##### Inclusion

We included articles that employed data science methods (machine learning, AI, big data analytics) to address MNCH indicators in Africa (including from African populations) and described their analytical approaches and performance. Peer reviewed articles and grey literature were included. 

##### Exclusion

Articles in languages other than English, as well as systematic reviews, scoping reviews, books, book chapters, studies without clear data science methodology and those not focussed on MNCH outcomes were excluded.

#### Data extraction and analysis

Data extraction followed the Preferred Reporting Items for Systematic Reviews and Meta-Analyses extension for scoping reviews (PRISMA-ScR) (Supplementary Table 2) [31]. Data extraction was performed independently by two reviewers using standardised forms developed priori. One reviewer extracted data from the peer-reviewed articles and using Rayyan software [32] for data management. The second reviewer focussed on extracting and analysing data from the grey literature. Discrepancies were resolved through discussion. The data we extracted included characteristics such as author, year, organisation, population, country, donor, study aims and objectives, study design or methods and approaches used, context, intervention (type, duration, recipients), key concepts and comparisons made and main findings. The extracted data were collated in Microsoft Excel [33].

### Section 1b: thematic analysis of scoping review: case studies and landscape overview

To provide a nuanced understanding of the practical application and impacts of data science in MNCH in Africa, we conducted an in-depth review of illustrative case studies. Additionally, we conducted a landscape overview to place these case studies within the broader context of data science in Africa using our published seven domains outlined in our conceptual framework (Figure S1) [4], which encompasses infrastructure and systemic challenges, data quality, governance and policy, technological innovations, capacity development, collaborations, and recommendations for implementation and scaling. The case study analysis aimed to highlight successful applications, identify common challenges and barriers, and showcase innovative approaches to leveraging data science for MNCH improvement in Africa.

### Sections 2a-2b: overview and thematic analysis of who’s digital health initiatives atlas

The third reviewer assessed projects registered on WHO’s Digital Health Initiatives Atlas [34] from 1970-to-2024 that focussed on MNCH in Africa. The projects were identified using keywords such as “pregnancy”, “breastfeeding”, “childbirth”, “birth registration”, “antenatal care”, “child health”, “family planning” and “maternal health”. Projects with MNCH components were classified as an MNCH Data Science initiative. We analysed data to identify countries leading in Data Science utilisation for healthcare generally and MNCH. The analysis focussed on the project’s overall background, involvement of donors, financial support from the government, use of software programmes, and challenges in carrying out the project. Descriptive statistics were conducted for analysis. We reviewed and synthesised the data using our MNCH data science framework with seven domains. All data analyses were conducted using Stata version 18.0 [35], Microsoft Excel 365 [33] and Quantum GIS version 3.36.2 [36].

## Results

**Fig. 1:**
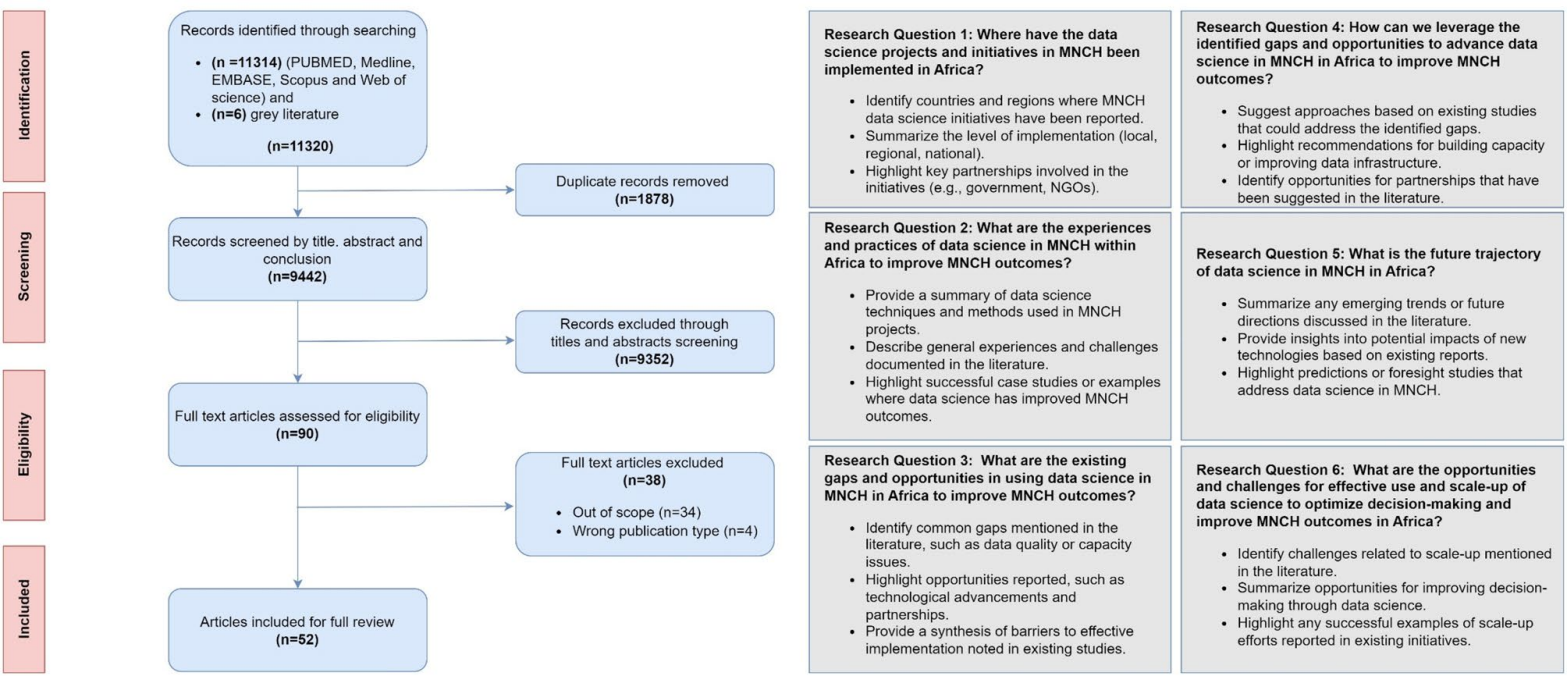
Overview of the study selection process and research questions. PRISMA flow diagram showing the scoping review research questions and methodology for identifying and selecting studies on data science applications in maternal, newborn, and child health (MNCH) in Africa. The process began with 11,320 records (11,314 records identified through database searching and six grey literature). Fifty two articles were included for full review after systematic screening and exclusion criteria were applied. The six research questions addressed by the review are shown on the right, covering implementation patterns, experiences and practices, gaps and opportunities, leveraging identified opportunities, future trajectories, and scale-up challenges for data science in MNCH across Africa

This scoping review addressed six research questions as outlined in our published protocol and Fig. 1: (1) Where have data science projects and initiatives in MNCH been implemented in Africa? (2) What are the experiences and practices of data science in MNCH within Africa to improve MNCH outcomes? (3) What are the existing gaps and opportunities in using data science in MNCH in Africa? (4) How can we leverage identified gaps and opportunities to advance data science in MNCH in Africa? (5) What is the future trajectory of data science in MNCH in Africa? (6) What are the opportunities and challenges for effective use and scale-up of data science to optimise decision-making and improve MNCH outcomes in Africa?

### Section 1a: overview of results from the scoping review

#### Selection of sources and evidence

We conducted a comprehensive literature search across five major databases: PubMed, MEDLINE, EMBASE, Scopus, and Web of Science, and grey literature resulting in 11,320 hits. After removing 1,878 (16.6%) duplicates, 9,442 articles remained for screening by title and abstract. Of these, 9,352 (99%) articles were excluded for failing to meet the inclusion criteria, primarily due to lack of focus on MNCH or did not have a data science focus. The remaining 90 articles (< 1%) underwent full text review. Of these, 38 articles were excluded–for being out of scope (*n* = 34) and four for being the wrong publication type (e.g., opinion pieces)–resulting in 52 articles included in the final scoping review (Fig. 1).

#### Characteristics of included studies

The 52 studies reviewed were published between 2016 and 2024 and increased each year from 2016 to 2024 (Supplementary Figures S2a and S2b). Most MNCH data science related studies conducted in Africa were not led by African authors, notably 13 studies (25.5%) had first author from the USA, followed by Ethiopia with 12 studies (23.5%). Other countries included Tanzania with 4 studies (7.8%), the UK with 3 studies (5.9%), and Ghana, India, Kenya, Uganda, and Zambia, each contributing 2 studies (3.9%). An additional 9 studies (17.6%) were contributed by authors from a range of other non-African countries, collectively grouped as “Others” (Table 1).

**Table 1:** Summary characteristics of included studies (n=52)

Study characteristics	Number of studies n (%)	Study References
First Author's Country	n=52	
USA	13 (25.0%)	[37–49]
Ethiopia	12 (23.1%)	[50–61]
Tanzania	4 (7.7%)	[62–65]
Uganda	3 (5.8%)	[66–68]
Ghana	3 (5.8%)	[69–71]
India	2 (3.8%)	[72, 73]
Kenya	2 (3.8%)	[74, 75]
UK	2 (3.8%)	[76, 77]
Zambia	2 (3.8%)	[78, 79]
Others*	9 (17.3%)	[80–88]
Regions of countries where studies were conducted	n=52	
Eastern: Zanzibar, Ethiopia, Zimbabwe, Uganda, Rwanda, Tanzania, Kenya, Zambia, Malawi	34 (65.4%)	[37, 40–44, 50–68, 72, 75, 77, 79, 82, 84, 86–88]
Multiple countries and regions: Tanzania, Zambia, Bangladesh, Pakistan, Ghana, Angola, Benin, Burkina Faso, Cameroon, Democratic Republic of the Congo, Cote d'Ivoire, Guinea, Lesotho, Madagascar, Malawi, Mali, Mozambique, Niger, Nigeria, Rwanda, Senegal, Zimbabwe, Chad, South Africa, Sierra Leone, Mauritania, Liberia, Gambia, USA	11 (21.2%)	[39, 45–48, 73, 74, 76, 83, 85]
Southern: South Africa	2 (3.8%)	[49, 78]
Western: Ghana	3 (5.8%)	[69–71]
Northern: Morocco	1 (1.9%)	[80]
Funding	n=52	
Funded	27 (51.9%)	[37–39, 41, 42, 44, 47, 48, 51, 52, 55, 62–67, 72–74, 76, 78, 80, 82, 83, 87, 88]
Not Funded	15 (28.8%)	[40, 43, 53, 54, 56, 58, 60, 61, 69–71, 75, 79, 81, 85, 86]
Not Indicated	10 (19.2%)	[45, 46, 49, 50, 57, 59, 68, 77, 84]
Data Source	n=52	
Study	28 (53.8%)	[37, 38, 41, 42, 44–47, 49, 50, 62, 64, 65, 67–70, 72, 73, 75–78, 80–82, 88]
Demographic Health Survey (DHS)	22 (42.3%)	[39, 40, 43, 48, 51–61, 66, 74, 79, 83–85, 87]
Multiple Indicator Cluster Survey	1 (1.9%)	[63]
Short Term Government Programme	1 (1.9%)	[86]
MNCH Thematic Area	n=52	
Child Health and Morbidity	8 (15.4%)	[37, 38, 50, 66, 67, 76, 80, 81]
Neonatal and Perinatal Mortality	6 (11.5%)	[51–53, 62, 74, 82]
Maternal and Child Mortality	5 (9.6%)	[39, 40, 69, 83, 84]
Estimation of gestational age	5 (9.6%)	[41, 42, 72, 73, 77]
Maternal and Child Nutrition	5 (9.6%)	[43, 54, 55, 70, 85]
Maternal Health and Morbidity	5 (9.6%)	[44, 45, 56–58]
Child birth and delivery	5 (9.6%)	[46, 59, 63, 64, 86]
Preterm birth and birthweight	3 (5.8%)	[47, 60, 78]
Universal health coverage and service delivery	3 (5.8%)	[61, 71, 75]
Stunting	2 (3.8%)	[79, 87]
Vaccination	2 (3.8%)	[48, 68]
Others (RMNCHN, Pregnancy outcomes and Ultrasound image classification	3 (5.8%)	[49, 65, 88]

The reviewed studies were conducted in 31 African countries, across several African regions (defined by the United Nations geoscheme for regional classification). The Eastern Africa region had the largest representation (34 studies (65.4%, 9 countries). Studies involving multiple countries and regions constituted 11 studies (21.5%) from 29 countries (26 were African, two were Asian and one North American countries) (Table 1). Twenty-seven (52%) studies were funded, whereas fifteen (29%) reported no external funding and ten studies (19%) did not indicate any funding information (Table 1 and supplementary Table S3). The majority of the reviewed articles were either studies (53.8%, *n* = 28) or national demographic health surveys (DHS) (42.3%, *n* = 22) rather than operational implementation in routine health systems (Table 1).

#### Focus on MNCH thematic area and outcomes

Most articles included in our review assessed various MNCH outcomes, notably child health and morbidity (15.4%, *n* = 8) [37, 38, 50, 66, 67, 76, 80, 81], neonatal and perinatal mortality (11.5%, *n* = 6) [51–53, 62, 74, 82], maternal and child mortality (9.6%, *n* = 5) [39, 40, 69, 83, 84], estimation of gestational age (9.6%, *n* = 5) [41, 42, 72, 73, 77], maternal and child nutrition (9.6%, *n* = 5) [43, 54, 55, 70, 85], maternal health and morbidity (9.6%, *n* = 5) [44, 45, 56–58], childbirth and delivery (9.6%, *n* = 5) [46, 59, 63, 64, 86], preterm birth and birthweight (5.8%, *n* = 3) [47, 60, 78], universal health coverage and service delivery (5.8%, *n* = 3) [61, 71, 75], stunting (3.8%, *n* = 2) [79, 87], vaccination (3.8%, *n* = 2) (i.e., DPT for Mali and Nigerian study and not specified for Ugandan Study) [48, 68], pregnancy outcomes (1.9%, *n* = 1) [49], reproductive, maternal, newborn, child health and nutrition (RMNCHN) (1.9%, *n* = 1) [65] and Ultrasound image processing (1.9%, *n* = 1) [88] (Tables 1 and 2).

**Table 2:** Main characteristics of included studies on use of data science in MNCH (n=52)

First Author	Year	Journal	First Author’s country	Article type	Outcome	MNCH thematic areas	Study Aim	Analytical methods used**	Data source	Funding Source	Study references
Nasejje & Mwambi	2022	BMJ	South Africa	Journal Article	u5MR	Maternal and child Mortality	predict U5MR	Random Forest, Neural Network	DHS	sub-Saharan Africa Consortium for Advanced Biostatistics training (SSACAB) grant as part of the DELTAS Africa Initiative	[83]
Mboya	2020	BMJ	Tanzania	Journal Article	perinatal death	Neonatal and Perinatal Mortality	determine key predictors of perinatal death	Random Forest, Neural Network, Naïve Bayes, Bagged Trees	Study	GSK Africa Non-Communicable Disease Open Lab through the DELTAS Africa Sub-Saharan African Consortium for Advanced Biostatistics (SSACAB)	[62]
Robi & Sitote	2023	Hindawi	Ethiopia	Journal Article	neonatal infections	Child Health and morbidity	To apply a classification stacking model for the following four main neonatal diseases: sepsis, birth asphyxia, necrotizing enterocolitis (NEC), and respiratory distress syndrome	Random Forest, Extreme Gradient Boosting, Support Vector Machine	Study	Not indicated	[50]
Ndagijimana	2023	JPMPH	Rwanda	Journal Article	Stunting	Stunting	To develop a prediction model for stunting	Random Forest, Extreme Gradient Boosting, Support Vector Machine, Naïve Bayes	DHS	World Bank funding (ID: ESC 91) through African Centre of Excellence in Data Science, University of Rwanda	[87]
Dejene	2022	BMC	Ethiopia	Journal Article	Maternal Anaemia	Maternal Health and Morbidity	Predict level of anaemia among pregnant women	Random Forest, Extreme Gradient Boosting, Decision Trees, Cat Boost	DHS	None	[56]
Aung	2019	GHRP	Tanzania	Journal Article	RMNCHN	RMNCHN	characterize data visualization interpretation capacity	Data visualisation	study	Not indicated	[65]
Fredriksson	2022	Frontiers in Digital Health	Zanzibar	Journal Article	location of delivery	Delivery	Build a prediction model	Random Forest, Neural Network, LASSO Regression	Uzazi Salama government programme	None	[86]
Chilyabanyama	2022	MDPI	Zambia	Journal Article	Stunting	Stunting	Predict stunting among children	Random Forest, Extreme Gradient Boosting, Support Vector Machine	DHS	None	[79]
Awadh	2021		Kenya	Thesis	UHC	Universal health coverage and service delivery	Role of health information systems in obtaining universal health coverage	Data visualisation	Study	None	[75]
Tarimo	2021	BMJ Open	Tanzania	Journal Article	Labour induction	Delivery	To Identify predictors of labour induction	Random Forest, Neural Network, Naïve Bayes	Hospital Registry	Research on CDC-Hospital-Community Trinity Coordinated Prevention and Control System for Major Infectious Diseases, Zhengzhou University	[63]
Hazlett	2023	Nature	USA	Journal Article	Infant mortality	Maternal and child Mortality	To estimate risk for infant mortality	Random Forest, Extreme Gradient Boosting, Kernel Cross validation, Elastic-Net	DHS	Not indicated	[39]
Fenta	2021	BMC	Ethiopia	Journal Article	Under 5 undernutrition	Maternal and child nutrition	Predict under-five undernutrition	Random Forest, Neural Network, LASSO Regression, Ridge Regression	DHS	None	[54]
Espinosa	2023	Science Advances	USA	Journal Article	Preterm birth	Preterm birth and birthweight	To identify signatures for maternal covariates affecting preterm birth	Extreme Gradient Boosting	Study	Not indicated	[47]
Kebede	2023	BMC	Ethiopia	Journal Article	Contraceptive discontinuation	Maternal Health and Morbidity	To predict and determine the predictors for contraceptive discontinuation in Ethiopia	Random Forest, Extreme Gradient Boosting, Neural Network, Support Vector Machine, Naïve Bayes, k-Nearest Neighbours, AdaBoost	DHS	Not indicated	[57]
Khan & Yunus	2023	Nutrition	Malaysia	Journal article	under 5 malnutrition	Maternal and child nutrition	To develop a majority voting-based hybrid ensemble learning model to accelerate prediction accuracy of malnutrition data of under-five children	Random Forest, Extreme Gradient Boosting, Decision Trees, k-Nearest Neighbours	DHS	None	[85]
Tsai	2023	Heliyon	USA	Journal Article	algorithm vulnerability	Child Health and morbidity	To evaluate algorithm's vulnerability to adversarial attacks	Random Forest, Neural Network, LASSO Regression	Study	Harvard Data Science Initiative	[37]
Kananura	2022	PLOS	Uganda	Journal Article	Acute respiratory infections; diarrhoea	Child Health and morbidity	To identify predictors of pneumonia and diarrhoea	Random Forest, Extreme Gradient Boosting, LASSO Regression, Bagged Trees	DHS	Not indicated	[66]
Syed	2019	JAMA	USA	Journal Article	Disease classification	Child Health and morbidity	To develop a convolution neural network to enhance detection of pathologic morphological features comparing diseased to healthy duodenal tissue	Neural Network, LASSO Regression	Study	Not indicated	[38]
Ganguli and Wagner	2024	American Journal of Obstetrics & Gynecology	USA	Abstract	risk for caesarean delivery	Delivery	To develop an objective, individualized risk prediction model for caesarean delivery using machine learning (ML) models trained on routine prenatal data.	Extreme Gradient Boosting	study	Not indicated	[46]
Lee	2023	JAMA	USA	Journal Article	Gestational age	Estimation of Gestational age	To develop artificial intelligence (AI) models to estimate GA	Neural Network	study	Google LLC; Bill and Melinda Gates Foundation	[77]
The Alliance for Maternal and Newborn Health Improvement (AMANHI) Gestational Age Study Group and Aftab	2021	BMJ Global Health	India	Journal Article	Gestational age	Estimation of Gestational age	To develop and validate programmatically feasible and accurate approaches to estimate newborn gestational age (GA) in low resource settings	Random Forest, Extreme Gradient Boosting, Support Vector Machine	Study	Bill and Melinda Gates Foundation	[73]
Anku & Duah	2024	PLoS ONE	Ghana	Journal Article	under 5 malnutrition	Maternal and child nutrition	To use machine learning (ML) algorithms to predict undernutrition and identify its associated factors	Random Forest, Extreme Gradient Boosting, Support Vector Machine, LASSO Regression, Ridge Regression	study	None	[70]
Bekele	2022	BMC Medical informatics and Decision Making	Ethiopia	Journal Article	low birth weight	Preterm birth and birthweight	To predict LBW in Ethiopia	Random Forest, Extreme Gradient Boosting, Support Vector Machine, Decision Trees, Naïve Bayes, k-Nearest Neighbours	DHS	None	[60]
Biswas	2023	BMJ Global Health	USA	Journal Article	zero dose vaccination	Vaccination	To examine how well predictive algorithms can characterise an individual child’s risk of being ZD	Decision Trees, k-Nearest Neighbours, Ridge Regression	DHS	GAVI, the Vaccine Alliance	[48]
Bitew	2020	Journal of Population Sciences	USA	Journal Article	under 5 mortality	Maternal and child Mortality	To predict important under-five mortality risks in Ethiopia	Random Forest, k-Nearest Neighbours	DHS	None	[40]
Bitew	2022	Public Health Nutrition	USA	Journal Article	Under 5 undernutrition	Maternal and child nutrition	To estimate predictive algorithms for the determinants of childhood stunting	Random Forest, Extreme Gradient Boosting, Neural Network, k-Nearest Neighbours	DHS	None	[43]
Bogale	2022	BMC Medical informatics and Decision Making	Ethiopia	Journal Article	maternal health	Neonatal and Perinatal Mortality	To predict perinatal mortality based on maternal health status and health insurance service using homogeneous ensemble machine learning methods	Random Forest, Extreme Gradient Boosting, Cat Boost	DHS	University of Gondar research and community service vice president's office.	[51]
Demsash	2023	PLOS ONE	Ethiopia	Journal Article	childhood vaccination	Neonatal and Perinatal Mortality	To predict perinatal mortality based on maternal health status and health insurance service using homogeneous ensemble machine learning methods	Random Forest, Naïve Bayes, AdaBoost, J48 classifier algorithm	DHS	None	[53]
Dereje	2021	IEEE Xplore	Ethiopia	conference paper	neonatal mortality	Neonatal and Perinatal Mortality	To investigate risk factors and predict neonatal and infant mortality based on maternal data	Bagged Trees, AdaBoost	DHS	Not stated	[52]
Gebeye	2024	Nutrition methodology	Ethiopia	Journal Article	childhood nutrition	Maternal and child nutrition	To identify important predictors of MN deficiency among children aged 6–23 months in Ethiopia using machine learning algorithms.	Random Forest, Neural Network, Support Vector Machine, Naïve Bayes	DHS	Wollo University research and community service vice president’s office.	[55]
Gough	2021	eBioNedicine	USA	Journal Article	gestational age, birth weight, neonatal growth	Estimation of Gestational age	To predict gestational age, birth weight and neonatal growth in rural Zimbabwe	Extreme Gradient Boosting	study	Bill and Melinda Gates Foundation Department for International Development, Wellcome Trust, Swiss Agency for Development and Cooperation, US National Institutes of Health, and UNICEF	[42]
Jeddi	2021	Healthcare (Basel)	Morocco	Journal Article	childhood asthma	Child Health and morbidity	to predict the occurrence of childhood asthma using data from a prospective study of 202 children with and without asthma	Support Vector Machine, Decision Trees	study	Belgium Ministry of cooperation through the VLIR UOS programme	[80]
Kovacs	2021	BMC pediatrics	Scotland	Journal Article	neonatal mortality	Neonatal and Perinatal Mortality	to provide a tool that provides clinically relevant cut-offs for predicting mortality that is easily used by clinicians in a low-resource setting	Decision Trees	study	Antimicrobial Resistance Cross-Council Initiative through a grant from the Medical Research Council; National Institute for Health Research	[48]
Kwizera	2019	Pediatric Critical Care Medicine	Uganda	Journal Article	childhood mortality	Child Health and morbidity	To deploy machine learning tools (random forests) to develop a model that reliably predicts hospital mortality in children with acute infections	Random Forest	study	Not indicated	[67]
Lee	2023	NPJ Digital medicine	UK	journal Article	fetal gestational age	Estimation of Gestational age	to estimate gestational age using only image analysis of standard ultrasound planes, without any measurement information.	Neural Network	study	Not indicated	[41]
Li	2023	European Journal of Medical Research	China	journal Article	childhood cerebral malaria	Child Health and morbidity	to study the molecular biological functions, signalling pathway changes and biological targets in the process of CM infection	Support Vector Machine, LASSO Regression	study	None	[81]
Mamo	2024	BMC Women's Health	Ethiopia	Journal Article	unintended pregnancy	Maternal Health and Morbidity	to assess the effectiveness of machine learning algorithms in predicting unintended pregnancy in Ethiopia and to identify the key predictors	Random Forest, Extreme Gradient Boosting, Decision Trees, Extra Tree classification	DHS	None	[58]
Mbunge	2023	IEEE Xplore	Zimbabwe	Conference paper	child mortality	Maternal and child Mortality	To use machine learning techniques to predict child mortality	Random Forest, Extreme Gradient Boosting, Decision Trees	DHS	Not indicated	[84]
Mulugeta	2023	IEEE Xplore	Ethiopia	conference paper	delivery date	Delivery	Determine key predictors of perinatal death	Random Forest, Neural Network	DHS	Not indicated	[59]
Murnane	2021	Journal of acquired immune deficiency syndrome	USA	journal Article	viremia	Maternal Health and Morbidity	To predict viremia to facilitate targeted adherence support in sub-Saharan Africa	LASSO Regression	study	Not indicated	[45]
Nareeba	2020	International Journal of Infectious Diseases	Uganda	journal Article	childhood immunization	Vaccination	To understand the determinants of childhood immunization in a rural Uganda using a machine learning method of Classification and Regression Tree	Decision Trees	study	Not indicated	[68]
Naydenova	2016	Journal of the royal society interface	UK	journal Article	childhood pneumonia	Child Health and morbidity	To develop a suite of data mining tools that facilitate automated diagnosis through quantifiable features	Random Forest, Support Vector Machine	study	EPSRC and the RCUK Digital Economy Programme, Skoll Centre for Social Entrepreneurship at the University of Oxford, Wellcome Trust	[76]
Nti & Owusu-Boadu	2022	Smart Health	Ghana	journal Article	maternal mortality	Maternal and child Mortality	To establish an intelligent machine learning-based system for effectively monitoring and predicting pregnant women's risk levels	Extreme Gradient Boosting	Study	None	[69]
Ogallo	2021	AMIA Annu Symp Proc	Kenya	journal Article	neonatal mortality	Neonatal and Perinatal Mortality	identifying the factors associated with neonatal mortality	Decision Trees	DHS	Bill & Melinda Gates Foundation	[74]
Petersen	2023	Paediatric and Perinatal Epidemiology	USA	journal Article	adverse pregnancy outcomes	Pregnancy ouctomes	to investigate associations between co-occurring placental features and adverse pregnancy outcomes in a prospective cohort of singletons	k-Nearest Neighbours	Study	Not indicated	[49]
Rittenhouse	2019	PLOS ONE	Zambia	journal Article	identification of preterm newborn identification	Preterm birth and birthweight	to leverage machine learning algorithms incorporating maternal factors associated with SGA to improve accuracy of preterm newborn identification in LMIC settings	Data visualisation	study	Bill and Melinda Gates Foundation grant to the Global Alliance to Prevent Prematurity and Stillbirth	[78]
Sazawal	2022	Journal of global health	India	journal Article	Gestational age	Estimation of Gestational age		Data visualisation	study	Bill & Melinda gates foundation	[72]
Sendra-Balcells	2023	Nature scientific reports	Spain	journal Article	fetal ultrasound	Ultrasound image processing	investigating the transferability of AI models trained in high-income settings and their applicability to process images acquired in low-income settings	Neural Network	study	European Union's 2020 research and innovation programme	[88]
Shah	2023	Frontiers in Digital Health	USA	Journal Article	postpartum hemorrhage	Maternal Health and Morbidity	to predict the occurrence of postpartum hemorrhage using machine learning models based on antenatal, intrapartum, and postnatal visit data	Random Forest, Decision Trees, Naïve Bayes	study	Bill & Melinda Gates Foundation	[44]
Tarimo	2021	Risk Management Healthcare policy	Tanzania	Journal Article	Apgar score	Delivery	to establish the most efficient boosting method in predicting neonatal low Apgar scores following labour induction intervention	Extreme Gradient Boosting	study	Research on CDC-Hospital-Community Trinity Coordinated Prevention and Control System for Major Infectious Diseases, Zhengzhou University	[64]
Tesfaye	2019	BMC Medical informatics and Decision Making	Ethiopia	journal Article	skilled delivery service use	Universal health coverage and service delivery	to identify determinants and develop a predictive model for skilled delivery service use in Ethiopia by applying logistic regression and machine-learning techniques	Neural Network, Support Vector Machine, Naïve Bayes	DHS	None	[61]
Boadu	2021	BioMed Research International	Ghana	Journal Article	healthcare delivery	Universal health coverage and service delivery	To assess healthcare providers attitude towards LHIMS system for service provision	Data visualisation	study	None	[71]

**Random Forest (n=28, 53.8%), Extreme Gradient Boosting (n=20, 38.5%), Neural Network (n=15, 28.8%), Support Vector Machine (n=12, 23.1%), Decision Trees (n=11, 21.2%), Naïve Bayes (n=9, 17.3%), LASSO Regression (n=8, 15.4%), k-Nearest Neighbors (n=7, 13.5%), Bagged Trees (n=3, 5.8%), AdaBoost (n=3, 5.8%), Ridge Regression (n=3, 5.8%), Other: Cat Boost, Kernel Cross validation, J48 classifier algorithm, Extra Tree classification, Elastic-Net and Data visualisation (n=11, 21.2%)

#### Data management, modelling and analytical approaches

The studies employed a broad array of machine learning approaches to support MNCH research. The most frequently used techniques included Random Forest (*n* = 28, 53.8%), Extreme Gradient Boosting (*n* = 20, 38.5%), Neural Networks (*n* = 15, 28.8%), Support Vector Machines (*n* = 12, 23.1%), Decision Trees (*n* = 11, 21.2%), Naïve Bayes (*n* = 9, 17.3%), the Least Absolute Shrinkage and Selection Operator (LASSO) regression (*n* = 8, 15.4%), k-Nearest Neighbors (*n* = 7, 13.5%), Bagged Trees (*n* = 3, 5.8%), AdaBoost (*n* = 3, 5.8%), Ridge Regression (*n* = 3, 5.8%), Other: Cat Boost, Kernel Cross validation, J48 classifier algorithm, Extra Tree classification, Elastic-Net and Data visualisation (*n* = 11, 21.2%) (Table 2).

### Section 1b: thematic analysis of results from the scoping review

In Africa, application of data science within MNCH reflects varied advancements and persistent challenges. We examined case studies according to seven domains previously defined in our published scoping review [4].

#### Domain I: infrastructure and systemic challenges

Most African countries have DHIS2, however, Mozambique [89, 90] and Kenya [91] have pioneered the deployment of the DHIS2 platform for national and subnational data aggregation, focusing on health indicators like preterm birth and low birth weight [89–91]. Most African countries utilise the DHIS2 platform to mostly capture aggregated data, although individual-level data collection is being explored [91]. Besides DHIS2, efforts in Nigeria [92, 93] and Ghana [94] have shown progress through development and utilisation of individual-level electronic health record systems, however, barriers such as fragmented digital ecosystems that work in silos with no unified plans for full national integration.

#### Domain II: data quality

While Mozambique and Kenya utilise DHIS2 primarily for aggregate data [89–91], Nigeria has experimented with patient-level data collection in tertiary hospitals, though this has not scaled nationwide [92, 93]. In Ghana, the Lightwave Hospital Information Management System (LHIMS) was introduced in 2017 to improve patient records management, however, challenges with consistent implementation due to electricity load shedding, poor internet connectivity, and inadequate logistics leads to a partial return to paper-based systems which affects data completeness [71], underscoring the need for enhanced digital infrastructure and policies supporting reliable data capture [94, 95].

#### Domain III: data governance, regulatory dynamics, and policy

Governance and policy frameworks differ significantly across the continent, influencing the governance of MNCH data science projects. Ghana’s e-Health strategy, introduced in 2010, aimed at strengthening data governance through a centralised LHIMS in all teaching hospitals in the country to improve patient records has encountered user acceptance issues that have limited its impact [71, 94, 95]. Similarly, Nigeria’s integration of patient-level data within tertiary hospitals demonstrates potential for data governance but requires broader policy support to reach nationwide implementation [92].

#### Domain IV: technological innovations and digital health

Several African countries are implementing unique digital tools to bridge healthcare gaps. For example, different digital tools were implemented in Ethiopia, Uganda, Tanzania, Kenya and Ghana (i.e., Enketo, ODK, RapidSMS, RapidPro, SMS, Native Android, Medic Mobile, CouchDB, iHRIS, Web, REDcap, LIMS (Laboratory Information Management System), SmartHIS, Postgres, and Python) (Table 3). The Western Cape in South Africa has created a digital health ecosystem, although the lack of a unified approach in other provinces in South Africa illustrates challenges in establishing a unified national electronic health record system [96–99].

**Table 3:** Summary of country case studies from the WHO’s digital health atlas

Attributes	Ethiopia	Nigeria	Kenya	Uganda	Tanzania	Malawi	Zambia	Ghana
Digital health attributes								
Number of projects	90	37	64	51	39	58	16	12
Number of MNCH focussed projects	11 (12%)	19 (51%)	34 (53%)	27 (53%)	29 (74%)	28 (48%)	11 (69%)	5 (42%)
Examples of project	safe delivery app to guide skilled health workers on Basic Emergency Obstetric and Neonatal Care, vital events registration, data collection	childhood vaccinations, pregnancy, birth and postpartum care, registration of births and health promotion, data collection.	eHealth and MNCH advice, digital payments, vaccinations, pregnancy registration and tracking ANC visits, and data collection	mHealth and MNCH trainings and advice, dashboards and visualisation, clinical decision making, communication and reminders, digital payments and MNCH data collection	mHealth and MNCH advice, vaccination, appointments, data collection	community-based decision support, pregnancy registration, communication, care-follow-up and referrals	MNCH registration and data collection, vaccinations, programme decision making, and communications	diagnostics, communication, vaccinations
Data science domains								
Domain I: Infrastructure & Systemic Challenges	Strong National coverage and international support (Izumi Foundation, Gates)	National-level coverage limited, individual-state projects	Strong county/national/multi-country coverage	Village to national implementation, strong telecom networks	Smallest units to national-level, multi-country projects	Government and donor-funded, multi-tiered implementation	Substantial donor support, government involvement	Government involved, some advancements in eHealth infrastructure
Domain II: Data Quality	Poor data quality, insufficient data utilization	Communication barriers, inadequate health worker motivation	Limited data utilization, high operational costs	Poor data quality, inadequate patient engagement	Poor guideline adherence, logistical issues	Delayed event reporting, poor health worker motivation	Poor data quality, health worker competence challenges	Poor data quality, insufficient worker competence
Domain III: Data Governance, Regulatory Dynamics, Policy	Strong government involvement in 10 (91%) of initiatives	Government supports 52% of projects	62% of projects funded by government	74% of projects funded by government	Government funds 62% of projects	64% government involvement	100% government-funded MNCH projects	80% government involvement
Domain IV: Technological Innovations & Digital Health	Use of IVR, DHIS 2, and OppiaMobile	AWS, Commcare, DHIS 2	DHIS 2, Community Health Toolkit, mobile/web apps	Commcare, Enketo, mobile platforms	Commcare, OpenSRP, SMS	MVTK, DHIS 2, mobile technologies	DHIS 2, OpenSRP, iHRIS, custom-built software	Blood Safety Info System, Carely Digital Health Platform
Domain V: Capacity Development, Human Capital, Opportunity	Substantial training of health workers	Training gaps, competence issues	Expanding capacity but hindered by high costs	Training and supervision gaps	Insufficient supervision of health workers	Delayed event reporting	Insufficient health worker competence	Limited health worker competence and engagement
Domain VI: Collaborative and Strategic Frameworks	International collaboration (WEEMA, Gates, etc.)	Collaboration with USAID, Gates	Collaboration with Grand Challenges Canada, CDC	Collaboration with Gates Foundation, CDC, Gavi	Collaboration with Gates, USAID, Gavi	Collaboration with Save the Children, World Bank, Skoll	Collaboration with UNICEF, DFID, Gates	Collaboration with CDC, Gates, DFID
Domain VII: Recommendations for Implementation & Scaling	Expand data utilization, improve service continuity	Improve health worker competence, better communication	Address operational costs, reach underserved areas	Address patient engagement, improve data quality	Improve supervision, address logistical issues	Improve event reporting, better health worker motivation	Address data quality, service delivery gaps	Improve health worker competence, expand eHealth solutions
Challenges	Poor data quality, service continuity issues	Health worker competence, communication barriers, motivation	Limited data use, operational costs	Poor patient engagement, data quality issues	Logistical challenges, inadequate supervision, motivation	Delayed reporting, low worker motivation	Poor data quality, health worker competence issues	Poor data quality, worker competence gaps

#### Domain V: capacity development, human capital, and opportunity

Kenya’s capacity development is advancing through initiatives such as the AMPATH programme, which supports workforce training across 300 + public health facilities [100, 101]. However, countries like Nigeria and Tanzania continue to face challenges related to training gaps and workforce motivation, constraining the effective application of data science in MNCH projects (Table 3).

#### Domain VI: collaborative and strategic frameworks

Collaborations underscore the role of external support in building robust data systems, for example, in Mozambique, DHIS2-SISMA was implemented through partnerships with international NGOs [89–91], while Ghana’s LHIMS and e-Health strategy benefited from collaborations with nursing and midwifery schools, WHO and other global health entities.

#### Domain VII: recommendations for implementation and scaling

Scaling and implementation of data science initiatives and innovations for MNCH requires strong resource allocation, governmental support, and adaptable technologies. Kenya and Uganda showcase scalable multi-level implementations of data science initiatives and DHIS2 systems to include all health facilities (public and private) and communities, but further scalability across the continent requires improved infrastructure, workforce development, and sustained funding to ensure broader impact in MNCH interventions.

###  Section 2a: overview of results from the WHO’s digital health initiatives atlas database

**Fig. 2:**
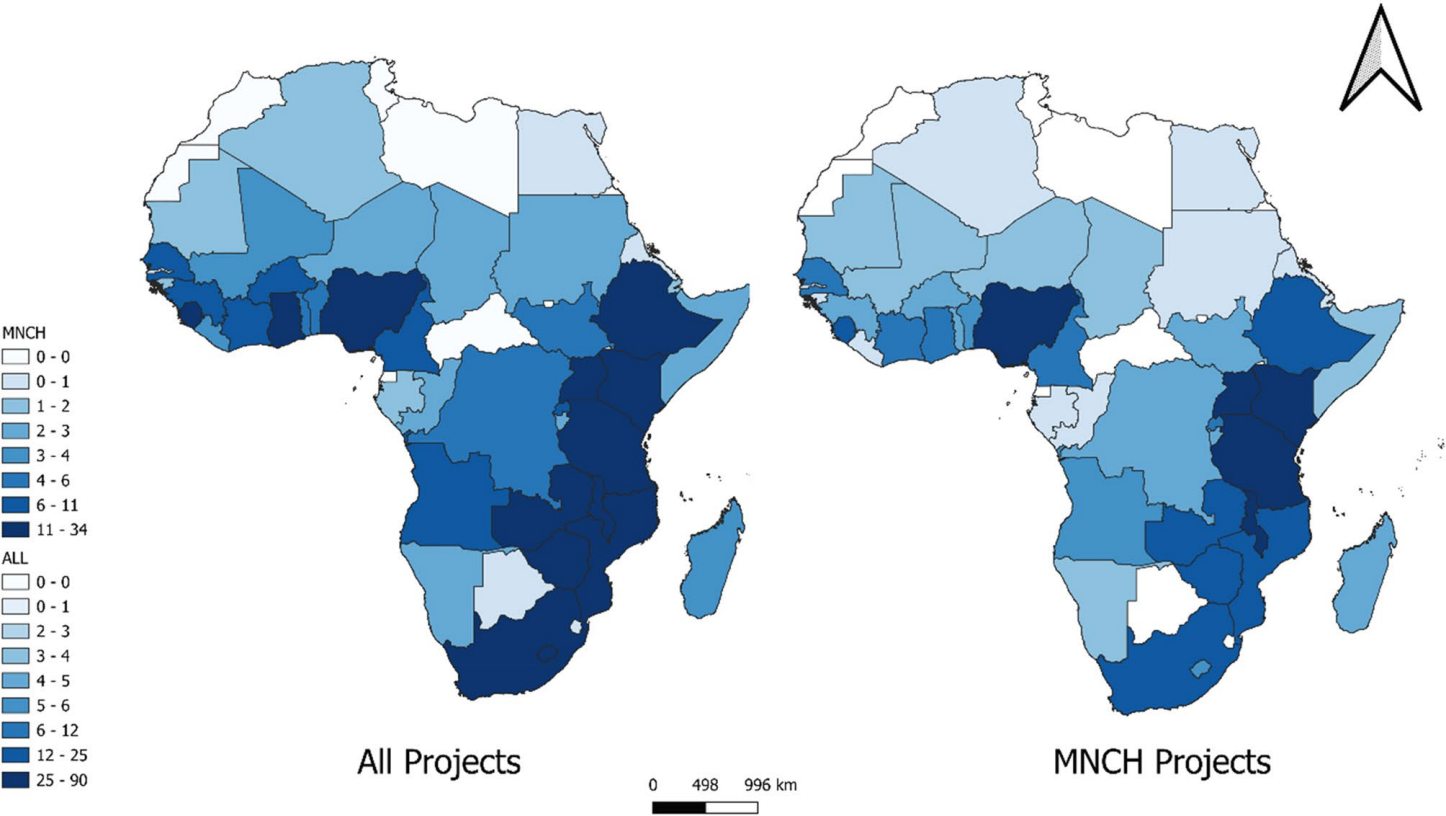
Distribution of data science initiatives in Africa. Geographic distribution of research projects across Africa, comparing all identified projects (left map) with MNCH-specific projects (right map)

We identified 659 data science projects implemented across 54 African nations, with 316 (48%) of these initiatives focussed on MNCH. These MNCH-focussed projects were implemented in 44 (82%) African countries Table 3, Supplementary Table S3 and Fig. 2.

The first recorded data science initiatives, both MNCH and Non-MNCH in Africa were launched in 2002, whereas similar initiatives outside Africa have been documented as far back as the 1970’s (Supplementary Table 3) The WHO Digital Health Atlas database had data on several aspects of MNCH including antenatal and postnatal care, childbirth and skilled birth attendance, maternal mortality and family planning services. For newborns, it included data on essential neonatal care practices, effective breastfeeding promotion, newborn mortality and stillbirths, and interventions to prevent mother-to-child transmission of HIV. Child health initiatives included immunisation coverage, managing common childhood illnesses, child and infant mortality and supporting nutrition and developmental milestones. Additionally, health systems strengthening themes included data collection, training health workers, and ensuring access to essential medicine.

###  Section 2b: thematic analysis of results from the who’s digital health initiatives atlas database

Using our published conceptual framework [4], we summarise countries with at least 10 projects in the WHO Digital Health Initiatives Atlas i.e., Ethiopia, Nigeria, Kenya, Uganda, Tanzania, Malawi, Zambia, and Ghana. While Ghana, implemented fewer projects overall, it has also made notable strides in digital health for MNCH, therefore was included in our assessment (Table 3 and Supplementary Table 3). The integration of data science into MNCH initiatives in the eight country case studies highlights advancements and persistent challenges within healthcare delivery (Table 3 and Supplementary Table 3).

#### Domain I: infrastructure and systemic challenges

All countries leverage a mix of donor support and existing digital health infrastructure to implement MNCH data science initiatives. Technologies like DHIS2 are widely used across Ethiopia, Kenya, Uganda, Tanzania and Zambia, allowing for structured data collection and management. However, systemic challenges, such as funding gaps and limited scalability of platforms persist. For example, Ethiopia benefits from strong international support and substantial national coverage, including backing from organisations like the Gates Foundation, while Nigeria struggles with national-level coverage, as most projects operate at state or regional levels. Similarly, Kenya benefits from a robust infrastructure with national and multi-country initiatives, but faces challenges in operational costs, which limit scalability. Uganda, Tanzania, Malawi, and Zambia have implemented initiatives at multiple administrative levels, from village to national and multi-country levels, but continue to grapple with logistical challenges in scaling these efforts. Ghana is progressing in eHealth infrastructure but still faces scalability issues due to existing systemic constraints (Table 3 and Supplementary Table 3).

#### Domain II: data quality

Data quality is a common issue across all countries. Poor data quality limits the potential of MNCH initiatives to yield actionable insights. Ethiopia, Nigeria, Uganda, and Ghana particularly struggle with insufficient data utilisation and quality concerns. Kenya faces high operational costs and limited data use, while Tanzania and Malawi contend with poor guideline adherence and delayed reporting. Zambia and Ghana both face issues with poor data quality and worker competence (Table 3 and Supplementary Table 3).

#### Domain III: data governance, regulatory dynamics, and policy

Government support is crucial in the governance of MNCH projects, with all countries receiving varying degrees of government backing. Zambia shows strong governmental commitment, with full funding support for its initiatives. Ethiopia and Uganda also demonstrate significant national support, while Kenya and Tanzania benefit from moderate government backing. Nigeria and Ghana are progressing toward stronger regulatory frameworks, but still require increased policy integration (Table 3 and Supplementary Table 3).

#### Domain IV: technological innovations and digital health

Technologies such as District Health Information Software version 2 (DHIS2), Commcare, and mobile health platforms are widely used across all countries to facilitate data collection, management and healthcare delivery, therefore, bridging the technological gap in healthcare access. All countries operate other digital technologies unique to them such as IVR and OppiaMobile in Ethiopia, AWS in Nigeria, REDCap, OpenSRP and SMS-based platforms in Uganda, Tanzania and Kenya. Ghana applies similar technologies in addition to advanced tools like the Blood Safety Information System (Table 3 and Supplementary Table 3).

#### Domain V: capacity development, human capital, and opportunity

Capacity building remains a significant challenge in many countries. Nigeria and Tanzania struggle with training gaps and health worker motivation, while Kenya faces high operational costs. Uganda and Malawi suffer from inadequate supervision and delayed reporting, respectively. Zambia and Ghana need to improve health worker competence to advance MNCH initiatives (Table 3 and Supplementary Table 3). These workforce-related barriers hinder the effective implementation and scaling of MNCH initiatives.

#### Domain VI: collaborative and strategic frameworks

Collaboration with international donors and organisations are central to MNCH initiatives’ success. All countries receive significant support from organisations (like the Gates Foundation, Gavi, DFID, World Bank Group and UNICEF) and research universities, fostering strategic partnerships to drive improved MNCH healthcare delivery. Many of the data science projects implemented are as a result of external funding (Table 3 and Supplementary Table 3).

#### Domain VII: recommendations for implementation and scaling

Implementation scope varies across countries, from local to national and multi-country levels. Scaling MNCH initiatives is dependent on effective resource allocation, government involvement, and the adoption of suitable technologies. Kenya and Uganda have implemented initiatives at multiple levels, allowing for broader coverage and adaptability (Table 3 and Supplementary Table 3).

## Discussion

### Key findings

In our scoping review, we mapped and provided an overview of data science use cases with applications to MNCH in Africa. We comprehensively reviewed and analysed case studies to provide insights into the current landscape and identified gaps and potential opportunities for improving MNCH in Africa with data science. The scoping review included 52 studies and found that no article used nationwide routinely collected data. These results highlight a critical gap in utilisation of routinely collected MNCH data in data science and machine learning. The fact that no reviewed article used nationwide routinely collected MNCH data could be indicative of limited electronic health records in SSA, although there were a number of electronic health records databases that are used regionally or nationally in some African countries.

A review of the WHO’s Digital Health Atlas found limited use of individual-level data in national health systems and instead, the DHIS2 platform was widely adopted for data aggregation of MNCH indicators. Data quality and completeness were common and reported as persistent challenges faced across MNCH data systems in Africa. In addition, there was a lack of significant government commitment and especially in regulatory support for adopting and scaling MNCH data science initiatives. Instead, there was still a heavy reliance on external partnerships and funding.

Some of the notable persistent challenges we identified to hinder progress of data science applications within MNCH include: poor data quality, insufficient workforce competence, and logistical challenges. These issues need to be addressed to maximise the potential of data science in MNCH initiatives in Africa. Addressing these challenges through targeted interventions in capacity building, improved data governance, and enhanced collaboration will be crucial for sustaining progress and scaling successful initiatives.

This review has several key strengths. First, we leveraged data from WHO’s Digital Health Initiatives Atlas, a globally recognised platform that provides comprehensive information on data science initiatives, covering many years of project implementation across 80% of African countries. Second, our analysis employed robust methods and tools, ensuring a thorough examination of MNCH data science projects across the continent.

Like any other study, ours is not without limitations, First, our reliance on specific keywords to identify MNCH data science initiatives may have excluded relevant projects, such as those focussed on COVID-19 or Civil Registration and Vital Statistics, which could indirectly impact MNCH. This specificity may have led to underreporting. Second, there are potential data gaps due to variations in reporting efforts across countries and the broad timeframe (2002–2024). Older projects, which might have been less documented, could have skewed our dataset toward more recent initiatives. Furthermore, limitations in our dataset regarding data cleaning, accuracy, and completeness mean our findings depend on the reporting standards and consistency of the WHO’s Digital Health Initiatives Atlas, therefore, our reliance on the WHO Digital Health Atlas may exclude smaller, locally funded initiatives that were not formally registered. Third, our study is primarily descriptive, limiting our ability to draw causal inferences or assess the effectiveness of MNCH data science projects in Africa. This approach provides a broad snapshot rather than an in-depth analysis of factors influencing project success or challenges. Fourth, our inclusion of only English language studies may have introduced publication bias, potentially underrepresenting other research within Africa that is published in French, Portuguese and Arabic. Finally, because our findings rely on projects registered within the WHO Digital Health Initiatives Atlas, they may not fully represent smaller or locally funded projects that were not registered, affecting the generalisability of our conclusions.

### Challenges for data science initiatives for MNCH in Africa

#### Data stewardship: data science skill set and capacity

In Africa, limited data literacy among its population poses a significant barrier on efforts to harness the power of data science in MNCH [102, 103]. Often, healthcare providers (e.g., nurses, doctors) who are responsible for recording data at health facilities lack formal training in data management and analysis [65]. Additionally, routine healthcare data across the continent are rarely subjected to quality assessments by trained data management professionals, which compromises the reliability and usability of the collected data [104]. This shortcoming is indicative of limited data stewardship, therefore, underscores the need for targeted capacity-building initiatives [105]. To address this, universities could incorporate continuous updates to existing curricula and introduce new programmes focussed on data science and management, thereby cultivating a skilled workforce capable of upholding high standards in data stewardship across healthcare sectors in Africa [105].

#### Data use, access, and ownership

Access to MNCH data within Health Information Systems (HIS) in Africa should be extended beyond healthcare providers, academia and policymakers, reaching the general public as well. Evidence from developed countries suggests that enabling citizens to access their health data through existing HIS platforms can generate public health benefits, empowering individuals to engage more actively in their health management [106]. For Africa to strengthen data ownership, it must not only innovate and build new technologies but also adapt and localise global initiatives to fit its unique contexts [107]. Fostering a culture of data use is essential and must be accompanied by efforts to build robust capacities for data curation. Developing a national data infrastructure that supports an integrated, high-quality ecosystem is critical. Such infrastructure should accommodate initiatives of all scales, ensuring comprehensive coverage across the continuum of care and facilitating better health outcomes continent-wide [108].

#### Government regulation and policies

Africa’s adoption of artificial intelligence (AI) technologies has been gradual, with only a few countries, including Benin, Egypt, Ghana, Mauritius, Rwanda, Senegal, and Tunisia, developing a national AI strategy [109–111]. In contrast, 77.8% (*n* = 42) of African countries have established national eHealth strategy reflecting broader engagement with digital health initiatives. Regulatory approaches to Artificial Intelligence (AI) often centre on data protection acts or regulatory institutions rather than comprehensive AI-specific policies. This results in limited effective policy frameworks and funding dedicated to AI research across countries [111, 112]. While the need for regulation in AI is widely acknowledged, some argue that minimal regulatory intervention could benefit early-stage innovations, allowing for more rapid development. The success of mobile money platforms, such as Kenya’s M-Pesa [113] and Uganda’s mobile money, illustrate this concept, where limited initial regulation enabled accelerated growth as a financial technology innovation [114]. For African countries aiming to implement AI, particularly within sensitive areas such as MNCH data, tailored strategies that balance regulation with innovation support could prove advantageous. This approach would allow African nations to harness AI’s potential while safeguarding the ethical and privacy considerations critical to healthcare data [115].

### Opportunities for data science initiatives for MNCH within Africa

Based on our findings, the review highlights the strong potential for data science to improve MNCH across Africa, although challenges still exist in light of the opportunities that are available. Data science offers valuable tools to strengthen health systems, empower healthcare workers, and enhance patient outcomes. Africa’s distinct demographic profile, growing adoption of new technologies, adaptable regulatory environment, and it’s “latecomer advantage” in digital innovation present unique opportunities. If strategically developed, these factors could create a strong foundation for data science initiatives to address MNCH challenges and lead to meaningful improvements in health outcomes across the continent.

#### Demographic opportunity

Africa represents 14.4% of the World population, with a significantly youthful demographic–70% of the population in SSA are under 30 years old compared to an aging global population that is observed in high-income countries [116–118]. This demographic trend offers a unique opportunity for the continent to harness its demographic dividend [119]. Therefore, Africa’s younger population can potentially champion the adoption and application of data science in MNCH. In addition, the innovative potential and fresh perspectives of African youths can provide the continent with an advantage to overcome barriers facing development of health research on the continent [120].

#### Data infrastructure and health information systems

The District Health Information Software version 2 (DHIS2) has been adopted by majority of African countries and other low-middle income countries as a national health information system. Although we found majority of the countries use the DHIS2 platform for only aggregated data. Experiences and lessons from the COVID-19 pandemic show that it can also be used to support individual level data. This is an opportunity for integrating individual-level MNCH data within the existing infrastructure across many SSA countries [121]. Another opportunity is that beyond Oslo University (the developers of DHIS2), within the African continent expertise to further develop and customise DHIS2 for MNCH data within Africa is available through the Health Information Systems Programmes (HISP) network [122, 123]. For example, HISP network partners are present in Kenya [124], Uganda [125], Rwanda [126], Mozambique [127], South Africa [128], Tanzania [129], and Zimbabwe [125]. The HISP networks in Africa are comprised of an East and Southern Africa network and a West African network. The two regional blocks have signed a Memorandum of Understanding in a recent meeting to merge and form a Pan-African network of HISP networks. These HISP partners are available to support DHIS2 customisation for other countries that have limited capacity [125].

#### Data standardisation and quality

Data standardisation is essential for maximising the potential of pooled datasets in healthcare. Frameworks such as the International Classification of Diseases (ICD-11) have significantly advanced the standardisation of clinical diagnoses, enabling a unified classification system that supports data pooling across multiple sources, countries, and regions. This standardised approach opens opportunities for data science applications, especially when applied to individual-level data [130]. In some countries, such as Ethiopia, customised solutions like the National Classification of Disease (NCOD) have been developed. This adaptation addresses the challenge of non-existent or uncommon diseases in the global standards, ensuring that local health data are accurately captured and coded [131]. Application of data science approaches such as large language models suffers from lack of standardised metadata that are essential for their utilisation.

To address this, ongoing initiatives in Africa aim to establish Common Data Models (CDMs). For example, the Implementation Network for Sharing Population Information from Research Entities (INSPIRE) data hub employs the Observational Medical Outcome Partnership (OMOP) CDM to harmonise data from longitudinal population studies across Africa [132]. Also the INDEPTH Network’s maternal newborn health working group spearheaded the standardisation of definitions of key MNCH indicators and data within the INDEPTH Network [133, 134]. Other opportunities aligned with data science and AI applications with internationally accepted guidelines are the Findable, Accessible, Interoperable, and Reusable (FAIR) principles [135]. Adhering to FAIR principles in Africa’s health information systems could ensure that data is AI-ready and available for use at the point of care, enhancing the quality and applicability of health data across the continent.

#### Leveraging mobile technology surge in Africa

The rise of mobile technology across Africa has facilitated the development of software solutions ranging from disease-specific platforms like DHIS2 to broader management systems like OpenMRS and custom mobile apps for data collection and patient management. Despite these advancements, challenges such as limited data quality, healthcare workforce shortages, and logistical barriers continue to hinder implementation. Integrating data science into healthcare has the potential to transform maternal and child health outcomes yet realising these benefits will require addressing gaps in funding, staff training, and infrastructure. Sustained support from international donors, along with government backing, will be key to overcoming these obstacles. Africa’s expanding digital backbone and internet connectivity also provide substantial opportunities, as seen in the success of mobile financial platforms like M-Pesa. Mobile health interventions have already improved maternal and neonatal healthcare utilisation in low- and middle-income countries for example in Uganda and Senegal, where digital payments for MNCH services have been used to improve coverage and remuneration of health workers during immunisation and other health campaigns for polio [27, 136]. Rwanda through Zipline’s drone initiative has improved delivery of medicines to patients in hard to reach areas [137]. By enhancing mobile technologies with AI capabilities and applying insights from existing implementations, Africa could leverage mobile health to drive further improvements in healthcare delivery and access.

#### High disease burden that calls for interventions that use data science

Despite significant progress towards achieving SDG 3 and ENAP across SSA, huge health disparities and disease burdens remain, especially in underserved communities [138]. Emerging technologies present an opportunity to utilise the growing volume of data generated at points of care, supporting data-driven decision-making that can enhance healthcare delivery and patient outcomes. The rising demand for data-informed strategies aligns well with Africa’s youthful population, which holds tremendous potential for data science applications. By investing in skills development and robust data infrastructure, African countries can harness this demographic advantage to drive impactful MNCH interventions and address existing health inequities.

#### Greenfield advantage and latecomer advantage

Africa’s health systems, particularly in MNCH, largely operate through traditional, paper-based methods. This “greenfield” or latecomer advantage means that, unlike more digitised systems, there is minimal need for extensive research, training, retraining or the “unlearning” of outdated technology, allowing Africa to adopt and implement the latest and most effective technologies from the outset. This opportunity positions African health systems to leapfrog directly into using advanced data science tools and techniques. For instance, new systems could incorporate Generative AI powered by large language models (LLMs) like ChatGPT and Bard, which have made data science concepts more accessible to a broader audience. The potential of Generative AI is particularly promising for addressing MNCH and broader healthcare challenges, although it should be noted that current LLMs, such as ChatGPT, are not specifically designed for medical applications and therefore bring performance and safety considerations that require careful adaptation and oversight in healthcare [139].

### Future trajectory of data science within MNCH in Africa

#### Data stewardship programmes

Currently, Africa has limited capacity-building initiatives in data science to fully harness the potential of data in maternal, newborn, and child health (MNCH). Developing long-term and sustainable data stewardship programmes—ensuring responsible management and use of research data—is essential for advancing Open Science and clinical research on the continent. Effective data stewardship involves structured planning for data handling, addressing challenges in data management, accessibility, and reliability [140]. By investing in data stewardship programmes, African countries can create a foundation for enhanced use of data science tools and techniques, overcoming barriers in MNCH data quality and paving the way for impactful healthcare solutions.

#### Multidisciplinary approach

Addressing the knowledge gaps and establishing priorities in MNCH data science requires a multidisciplinary approach that involves active engagement across multiple sectors. Stakeholder involvement at the policy level, particularly in areas such as data use and protection, is essential for ensuring ethical and effective data application. Civil society organisations, private-sector partners, and public entities should be integral to these efforts, promoting private-public partnerships that transform research findings into actionable products and services. Observations from the current review suggest that research findings are often published without being translated into testable, real-world applications. By fostering collaboration among policymakers, implementation researchers, private-sector actors, and other stakeholders, there can be a stronger uptake and integration of data science solutions. Additionally, adopting citizen-centred design will help ensure that data science and AI tools are responsive to public health needs and well-suited to the African context.

#### Programmatic implementation at scale

To harness the full potential of data science in MNCH, strengthening Africa’s data systems is essential. The current reliance on aggregate data limits actionable insights, underscoring the need for more granular data systems capable of driving meaningful change. Our review reveals that most studies in Africa focus on foundational research in data science and AI for MNCH, rather than programmatic implementation. For greater impact, research efforts must be closely linked to implementation partners, start-ups, and the development of minimum viable products with scalability potential. Achieving this requires balancing ethical considerations, regulatory frameworks, and operational flexibility, while promoting grassroots data use and minimising data migration challenges.

### Next steps and recommendations

Our scoping review found various limitations which constrain the development and application of data science tools in MNCH. Effective use of data science in healthcare requires substantial investments in data creation, curation, and skilled personnel. However, many governments hesitate to invest without clear evidence of the benefits—creating a cycle in which data science applications lack the initial support needed to demonstrate their value in healthcare. Mobilising both local and international funding will be critical to building data stewardship capacity and supporting scalable health solutions. Africa’s latecomer advantage allows MNCH initiatives to leapfrog directly into advanced data science applications, leveraging the continent’s unique demographic landscape for broader impact. The rapid growth of mobile technology and the vast amounts of data it generates offer an unprecedented opportunity to build analytics capacity, addressing the region’s health challenges and improving MNCH outcomes in sub-Saharan Africa.

We make five key recommendations that could potentially help break this cycle, enabling African governments, institutions, and international partners to support data science for MNCH in Africa. By addressing these areas, Africa can build a robust data science ecosystem that advances MNCH and improves healthcare outcomes across the continent [141].

1. *Investment in Infrastructure*: Building data repositories and computational resources across Africa will create the foundation for advanced data science applications, facilitating more impactful MNCH initiatives.
2. *Training, Capacity Building, and Capacity Strengthening*: Expanding data science training programmes will upskill Africa’s young workforce, making data science initiatives more scalable and sustainable.
3. *Collaborations*: Partnerships within Africa, as well as with global and high-income country partners, can foster knowledge sharing, best practices, and technology transfer to accelerate data science advancements.
4. *Funding*: Increased investment from both African governments and philanthropy and international donors, including grants and loans, will drive the development of data science tools and evidence-based interventions to address MNCH needs on the continent.
5. *Governance and Ethics*: Clear frameworks for ethical data use are essential. Governments, researchers, and policymakers should prioritise ethical guidelines to ensure responsible implementation of data science in healthcare.

## Conclusion

This scoping review mapped the current landscape of data science applications in MNCH across Africa, revealing both opportunities and challenges. While 52 studies demonstrated various applications of machine learning and AI techniques, most remained research-focussed rather than operationally integrated. The WHO Digital Health Atlas showed 316 MNCH-focussed initiatives across 44 African countries, indicating growing interest but persistent challenges in data quality, infrastructure, and capacity. Africa’s demographic advantage, expanding digital infrastructure, and latecomer advantage in technology adoption present significant opportunities for advancing data science applications in MNCH.

## Supplementary Information

Supplementary Material 1

Supplementary Material 2

Supplementary Material 3

## Data Availability

All data generated or analysed during this scoping review are publicly available or included in this published article and its supplementary information files. The 52 articles that we have included in the scoping review are listed in Table 1 and the detailed supplementary materials. Data from the **WHO Digital Health Initiatives Atlas** are also publicly available and accessible through its original URL [https://digitalhealthatlas.org/en/-/](https:/digitalhealthatlas.org/en/-) or via “**Implementome**” it’s new host [https://gdhub.unige.ch/implementome/projects](https:/gdhub.unige.ch/implementome/projects). Any additional materials used in this study are available from the corresponding authors Professor Eric O. Ohuma ([Eric.Ohuma@lshtm.ac.uk](mailto:Eric.Ohuma@lshtm.ac.uk)) and Dr Akuze Joseph Waiswa ([joseph.waiswa@lshtm.ac.uk](mailto:joseph.waiswa@lshtm.ac.uk)) both at London School of Hygiene & Tropical Medicine, upon reasonable request.

## Abbreviations

AI: Artificial Intelligence
AMPATH: Academic Model Providing Access to Healthcare
CDM: Common Data Models
DHA: Digital Health Atlas
DHS: Demographic and Health Survey
DHIS2: District Health Information Software version 2
DPT: Diphtheria, Pertussis, Tetanus
DS-I Africa: Data Science for Health Discovery and Innovation in Africa
ENAP: Every Newborn Action Plan
EPMM: Ending Preventable Maternal Mortality
EWENE: Every Woman Every Newborn Everywhere
FAIR: Findable, Accessible, Interoperable, and Reusable
HIS: Health Information Systems
HISP: Health Information Systems Programmes
ICD 11: International Classification of Diseases 11
INSPIRE: Implementation Network for Sharing Population Information from Research Entities
LASSO: Least Absolute Shrinkage and Selection Operator
LHIMS: Lightwave Hospital Information Management System
LLMs: Large Language Models
m-health: Mobile health technology
MDGs: Millennium Development Goals
MICS: Multiple Indicator Cluster Surveys
MNCH: Maternal, Newborn and Child Health
NCOD: National Classification of Disease
OMOP: Observational Medical Outcome Partnership
OSF: Open Science Framework
PRISMA-ScR: Preferred Reporting Items for Systematic Reviews and Meta-Analyses extension for Scoping reviews
RMNCHN: Reproductive, Maternal, Newborn, Child Health and Nutrition
SDGs: Sustainable Development Goals
SSA: Sub-Saharan African
