# Supplementary material for: Data science and artificial intelligence for maternal, newborn and child health: scoping review and thematic analysis
Source: BMC Public Health. 2025 Nov 28;26:314. doi: 10.1186/s12889-025-25430-0 (PMC12838465; doi:10.1186/s12889-025-25430-0)
Supplement: Supplementary file 1 — Supplementary Material 1 [file 12889_2025_25430_MOESM1_ESM.docx]

Figure S1: MNCH Data Science scoping review conceptual framework


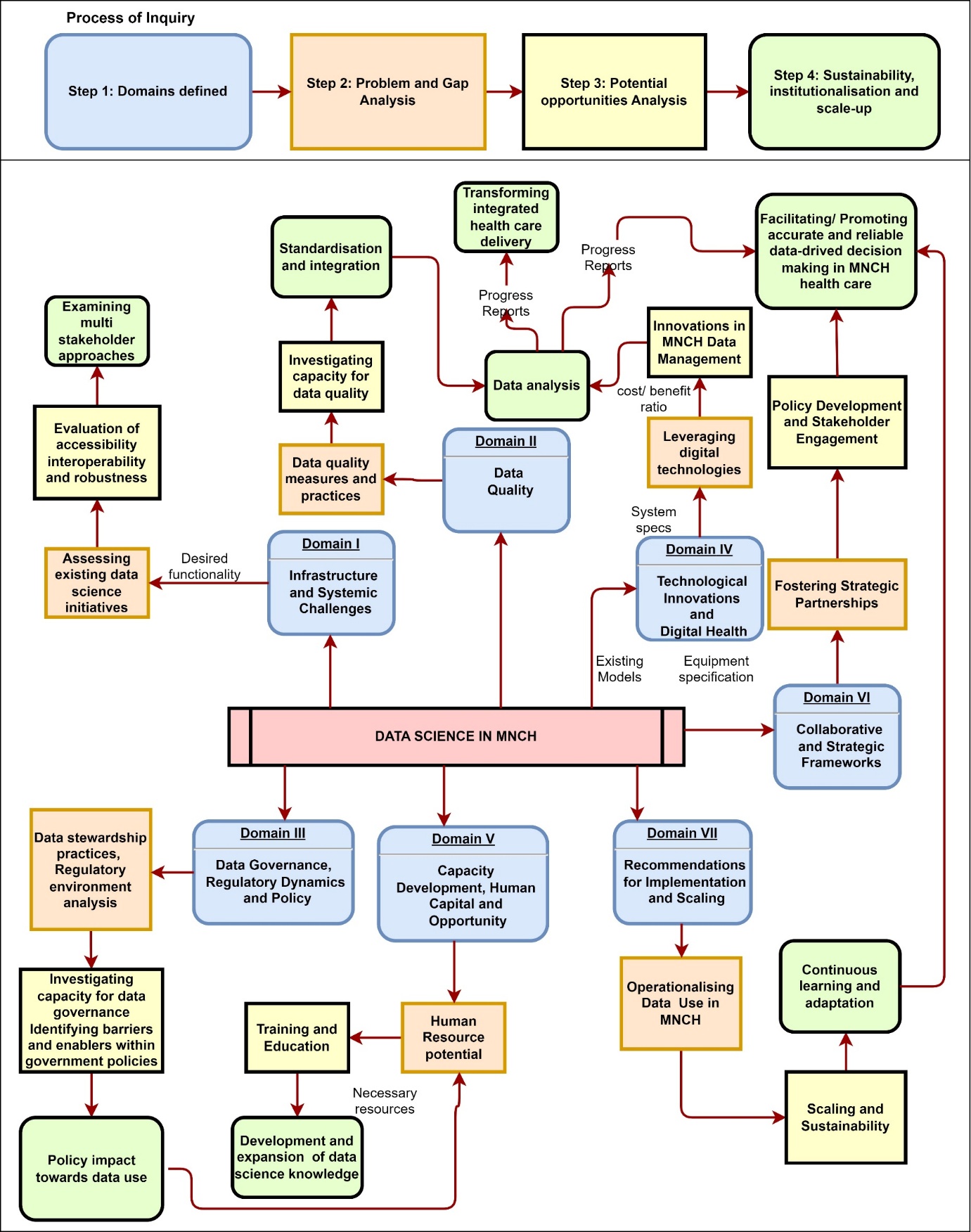


*Conceptual framework for harnessing data science role in improving MNCH in Africa. The conceptual model represents the process of inquiry for data science’s role in MNCH in Africa. In step 1, the main domains of inquiry represented with blue boxes are defined. Step 2 is the problem and gap analysis represented by orange boxes. Step 3 is the potential opportunities analysis represented by yellow boxes. Finally, step 4 will include sustainability, institutionalisation and scale-up potential for each domain.*

**Source**: Published by authors in BMJ Open <https://bmjopen.bmj.com/content/bmjopen/14/12/e091883.full.pdf>

Figure S2a: Number of articles reviewed

Figure S2b: Map of Africa showing the number of articles reviewed by country
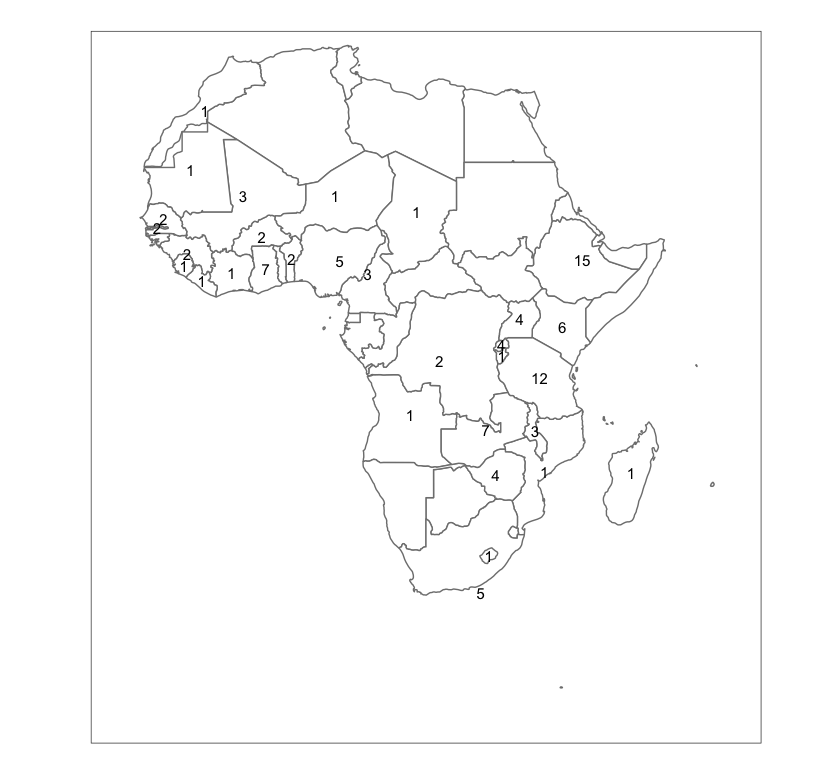


Table S1: Detailed search terms

| #1 | "Africa" OR "Algeria" OR "Angola" OR "Benin" OR "Botswana" OR "Burkina Faso" OR "Burundi" OR "Cameroon" OR "Cape Verde" OR "Central African Republic" OR "Chad" OR "Democratic Republic of Congo" OR "Republic of Congo" OR "Cote d'Ivoire" OR "Djibouti" OR "Egypt" OR "Equatorial Guinea" OR "Eritrea" OR "Ethiopia" OR "Gabon" OR "Gambia" OR "Ghana" OR "Guinea" OR "Guinea Bissau" OR "Kenya" OR "Lesotho" OR "Liberia" OR "Libya" OR "Madagascar" OR "Malawi" OR "Mali" OR "Mauritania" OR "Mauritius" OR "Morocco" OR "Mozambique" OR "Namibia" OR "Niger" OR "Nigeria" OR "Reunion" OR "Rwanda" OR "Sao Tome and Principe" OR "Senegal" OR "Seychelles" OR "Sierra Leone" OR "Somalia" OR "South Africa" OR "South Sudan" OR "Sudan" OR "Swaziland" OR "Tanzania" OR "Togo" OR "Tunisia" OR "Uganda" OR "Zambia" OR "Zimbabwe" OR "Southern Africa" OR "East Africa" OR "Central Africa" OR "Northern Africa" OR "West Africa" |
| --- | --- |
| #2 | (maternal[MeSH Terms])OR (neonatal[MeSH Terms]) OR (child[MeSH Terms]) OR perinatal |
| #3 | (("data science") OR ("artificial intelligence") OR (AI) OR ("machine learning")) |
| Final Search | #1 AND #2 AND #3 |
